# Diabetes Mellitus and In-Hospital Outcomes in Hospitalized COVID-19 Patients: A Single-Center Eastern European Cohort Study (2020–2024)

**DOI:** 10.3390/medicina62020410

**Published:** 2026-02-21

**Authors:** Ioana-Georgiana Cotet, Ana-Olivia Toma, Diana-Maria Mateescu, Adrian-Cosmin Ilie, Sorin Ursoniu, Dan Alexandru Surducan, Cosmin Gridan, Stela Iurciuc, Dragos-Mihai Gavrilescu, Cristina Tudoran

**Affiliations:** 1 Doctoral School, Department of General Medicine, “Victor Babes” University of Medicine and Pharmacy Timisoara, Eftimie Murgu Square No. 2, 300041 Timisoara, Romania; ioana.cotet@umft.ro (I.-G.C.); diana.mateescu@umft.ro (D.-M.M.); 2 Centre of Molecular Research in Nephrology and Vascular Disease, “Victor Babes” University of Medicine and Pharmacy Timisoara, Eftimie Murgu Square No. 2, 300041 Timisoara, Romania; tudoran.cristina@umft.ro; 3 Discipline of Dermatology, “Victor Babes” University of Medicine and Pharmacy Timisoara, Eftimie Murgu Square No. 2, 300041 Timisoara, Romania; toma.olivia@umft.ro; 4 Center for the Morphologic Study of the Skin (MORPHODERM), “Victor Babes” University of Medicine and Pharmacy Timisoara, 300041 Timisoara, Romania; 5 Department of Public Health and Sanitary Management, “Victor Babes” University of Medicine and Pharmacy Timisoara, Eftimie Murgu Square No. 2, 300041 Timisoara, Romania; ilie.adrian@umft.ro (A.-C.I.); sursoniu@umft.ro (S.U.); surducan.dan@umft.ro (D.A.S.); 6 Centre for Translational Research and Systems Medicine, Faculty of Medicine, “Victor Babes” University of Medicine and Pharmacy Timisoara, Eftimie Murgu Square No. 2, 300041 Timisoara, Romania; 7 Department of Orthopedics and Traumatology, Railway Clinical Hospital (CFR), Tudor Vladimirescu Street 13-15, 300173 Timisoara, Romania; 8 Cardiology Department, “Victor Babes” University of Medicine and Pharmacy Timisoara, Eftimie Murgu Square No. 2, 300041 Timisoara, Romania; 9 Department of Orthodontics, Dental District, Strada Zăgazului Nr. 3, ONE Floreasca Vista, Sector 1, 014261 Bucharest, Romania; 10 Department VII, Internal Medicine II, Discipline of Cardiology, “Victor Babes” University of Medicine and Pharmacy Timisoara, Eftimie Murgu Square No. 2, 300041 Timisoara, Romania; 11 County Emergency Hospital “Pius Brinzeu”, L. Rebreanu, Nr. 156, 300723 Timisoara, Romania

**Keywords:** COVID-19, SARS-CoV-2, diabetes mellitus, in-hospital mortality, intensive care unit admission, cardiovascular complications, thromboembolism, pulmonary embolism, Eastern Europe

## Abstract

*Background and Objectives*: Diabetes mellitus (DM) has been consistently linked to severe coronavirus disease 2019 (COVID-19) and adverse outcomes; however, the extent to which DM independently predicts mortality and cardiovascular complications in real-world hospitalized cohorts remains debated, particularly in Eastern Europe. This study aimed to evaluate the impact of DM on cardiovascular complications and in-hospital outcomes among adults hospitalized with SARS-CoV-2 infection. *Materials and Methods*: We conducted a single-center retrospective observational cohort study including consecutive adult patients hospitalized with laboratory-confirmed SARS-CoV-2 infection between March 2020 and December 2024 at the “Victor Babeș” Clinical Hospital of Infectious Diseases and Pneumophthisiology, Timișoara, Romania. DM status (type 1, type 2, or newly diagnosed diabetes) was defined using structured dataset fields. The primary outcome was in-hospital all-cause mortality. Secondary outcomes included ICU admission, length of stay, pulmonary embolism (PE) on CT pulmonary angiography (CTPA), and a composite of in-hospital cardiovascular/thromboembolic complications. Multivariable logistic regression models adjusted for clinically relevant covariates (age, sex, BMI, vaccination status, hypertension, ischemic heart disease, atrial fibrillation, prior ischemic stroke, and admission creatinine). *Results*: A total of 395 patients were included; 98 (24.8%) had DM. Diabetic patients exhibited a high cardiometabolic burden (arterial hypertension: 83.7% vs. 77.4%, *p* = 0.242) and higher admission renal markers (urea: 55.6 [41.0–79.1] vs. 48.6 [39.2–68.0] mg/dL, *p* = 0.047; creatinine: 1.04 [0.76–1.52] vs. 0.88 [0.59–1.33] mg/dL, *p* = 0.008). In-hospital mortality was numerically higher in DM (9.2% vs. 6.7%, *p* = 0.560), as was ICU admission (7.1% vs. 4.7%, *p* = 0.503), without statistical significance. PE on CTPA occurred in 13.3% of DM vs. 11.4% of non-DM patients (*p* = 0.763). In univariable analysis, DM was not significantly associated with mortality (OR 1.40, 95% CI 0.62–3.19; *p* = 0.422) or ICU admission (OR 1.55, 95% CI 0.61–3.97; *p* = 0.356). After multivariable adjustment, DM remained not independently associated with mortality (adjusted OR 1.09, 95% CI 0.42–2.83; *p* = 0.854) or ICU admission (adjusted OR 1.19, 95% CI 0.42–3.36; *p* = 0.747). *Conclusions*: In this real-world Eastern European cohort of hospitalized adults with SARS-CoV-2 infection, diabetes mellitus was common and associated with significantly worse renal function at admission, but it was not statistically associated with in-hospital mortality or ICU admission after multivariable adjustment; however, the limited number of events and low events-per-variable raise concerns about model stability and potential false-negative findings. These findings support a risk-marker model in which adverse COVID-19 outcomes in diabetic patients are driven primarily by clustered vulnerability and organ dysfunction rather than diabetes status alone.

## 1. Introduction

Coronavirus disease 2019 (COVID-19), caused by severe acute respiratory syndrome coronavirus 2 (SARS-CoV-2), rapidly evolved from an acute respiratory illness into a complex multisystem disorder. This disorder is characterized by immune dysregulation, endothelial injury, and thromboinflammatory activation. Early observational cohorts showed that adverse outcomes among hospitalized patients are driven not only by viral pneumonia, but also by host-related factors—particularly advanced age and pre-existing cardiometabolic comorbidities. Such factors substantially influence the risk of organ dysfunction and mortality [1,2].

Among cardiometabolic conditions, diabetes mellitus (DM) has consistently emerged as a major determinant of COVID-19 severity and mortality in diverse healthcare systems. Large population-based studies and linked electronic health record analyses have identified DM as an independent risk factor for COVID-19-related death, even after adjustment for demographic characteristics and associated comorbidities [2,3,4]. Within diabetic populations, COVID-19 outcomes appear to be strongly affected by the presence of cardiovascular disease, renal dysfunction, and overall cardiometabolic burden. This suggests a clinically relevant interaction between diabetes and cardiovascular vulnerability in SARS-CoV-2 infection [3,4]. Global umbrella meta-analyses further support these findings, consistently showing an increased risk of severe disease and mortality among patients with DM [5].

The pathophysiological mechanisms linking DM and poor COVID-19 outcomes go beyond hyperglycaemia alone. Diabetes is marked by chronic low-grade inflammation, impaired immune responses, endothelial dysfunction, and a prothrombotic state. It is often accompanied by hypertension, chronic kidney disease, and atherosclerotic cardiovascular disease [6]. During acute SARS-CoV-2 infection, these preexisting problems intersect with virus-induced endothelial injury, cytokine-driven inflammation, and coagulation activation. This intersection heightens the risk of microvascular dysfunction and cardiovascular complications [7,8]. Histopathological studies have shown diffuse endothelialitis, thrombosis, and microangiopathy in severe COVID-19. These provide a mechanistic basis for multiorgan injury, including the heart [7,8].

Cardiovascular complications are a defining feature of severe COVID-19 and a major cause of in-hospital morbidity and mortality. Clinical and mechanistic studies report high rates of myocardial injury, arrhythmias, heart failure decompensation, and thromboembolic events, especially in patients with pre-existing cardiovascular disease [9]. Myocardial injury, often measured by elevated cardiac troponin, is repeatedly linked to higher disease severity and mortality in hospitalized COVID-19 patients [10,11,12,13]. Although the causes of troponin elevation in COVID-19 vary—including supply–demand mismatch, inflammation, microvascular dysfunction, and acute coronary syndromes—cardiac biomarker elevation reliably indicates a worse clinical course. Cardiac troponin has therefore been widely adopted as a clinically actionable biomarker for risk stratification in hospitalized COVID-19 patients [14,15,16].

In patients with diabetes, cardiovascular involvement may directly link SARS-CoV-2 infection to poor outcomes. Multicenter observational studies show that hospitalized patients with DM have high rates of respiratory failure, ICU admission, and death. Risks are strongly influenced by age, diabetes complications, and the burden of cardiovascular disease [7]. Evidence also suggests that in-hospital metabolic control affects prognosis. Improved glycaemic control during hospitalization is linked to better outcomes in patients with pre-existing type 2 diabetes. This supports the interplay between metabolic stress, systemic inflammation, and cardiovascular injury during acute infection [6].

Despite growing international evidence, few clinically detailed hospital-based analyses specifically examine how diabetes and cardiovascular complications together affect in-hospital outcomes, particularly in real-world cohorts from Eastern Europe. Large-scale electronic health record analyses and nationwide diabetes cohorts have consistently documented excess COVID-19 mortality risk in patients with diabetes, while also underscoring the contribution of age and cardiometabolic/renal comorbidity burden [15,16]. Regional differences in baseline cardiometabolic risk, healthcare delivery, and clinical management highlight the need for local data. This study thus aimed to evaluate how diabetes affects cardiovascular complications and clinical outcomes in hospitalized patients with SARS-CoV-2. The focus is on in-hospital mortality, disease severity, and markers of cardiovascular injury. We hypothesized that diabetes status would be associated with worse crude outcomes but would lose independent significance after adjustment for age, cardiovascular comorbidity burden, and renal dysfunction.

## 2. Materials and Methods

### 2.1. Study Design and Setting

This single-center retrospective observational cohort study was conducted in the Infectious Diseases Department 1 of the “Victor Babeș” Clinical Hospital of Infectious Diseases and Pneumophthisiology, Timișoara, Romania. The study included all consecutive adult patients hospitalized with laboratory-confirmed SARS-CoV-2 infection between 1 March 2020 and 31 December 2024, reflecting a real-world, unselected population admitted during this predefined period. Clinical data were extracted from a standardized departmental Excel-based database, which is routinely used for prospective documentation of admission characteristics, inpatient clinical course, discharge outcomes, and institutional research reporting.

### 2.2. Study Population

Adult patients (≥18 years) hospitalized with a primary diagnosis of COVID-19 during the study period were eligible. SARS-CoV-2 infection at admission was confirmed through reverse-transcription polymerase chain reaction (RT-PCR) and/or validated antigen testing, as recorded in the database.

To avoid duplicate observations, only the first hospitalization per patient during the study interval was included as the index hospitalization. Patients were included if they had (i) a documented COVID-19 hospitalization within the study period and (ii) available in-hospital outcome information, including discharge status (alive or deceased).

Missing data were handled using an available-case approach (pairwise deletion), whereby patients with missing values were excluded only from analyses that required those specific fields. The key exposure and outcome variables were diabetes status (DM vs. non-DM) and in-hospital mortality, respectively.

### 2.3. Data Collection and Variables

All study variables were obtained from the departmental electronic dataset, which prospectively collects standardized information at admission, during hospitalization, and at discharge.

Age was recorded in years at admission. Sex was recorded as male or female, based on patient identification documents. Place of residence was categorized as urban or rural according to administrative records. COVID-19 vaccination status was defined as vaccinated if the patient had received any dose of a SARS-CoV-2 vaccine prior to admission and as unvaccinated otherwise. For the purposes of this analysis, vaccination status was modelled as a binary variable (any prior vaccination vs. none), because detailed information on dose number and booster status was inconsistently available. BMI was calculated as weight in kilograms divided by height in meters squared (kg/m^2^). Smoking status was specified as current smoker, former smoker, or never smoker, as documented in the database.

Baseline comorbidities were retrieved from dedicated structured fields. Diabetes-related data were obtained from diabetes-specific variables (type 1 diabetes, type 2 diabetes, newly diagnosed diabetes) and the diabetes/nutrition comorbidity domain. Cardiovascular history included arterial hypertension, ischemic heart disease, atrial fibrillation, prior ischemic stroke, left ventricular hypertrophy, and other cardiovascular conditions documented by treating physicians. Additional comorbidity domains recorded in the database (e.g., renal, pulmonary, gastrointestinal) were considered as potential covariates in adjusted analyses, provided they were clinically relevant and data completeness supported their inclusion.

Markers of clinical severity included the symptom-onset-to-admission interval (days), peripheral oxygen saturation (SpO_2_) at presentation, and the need for respiratory support. Respiratory support variables comprised oxygen requirement, type of oxygen therapy (e.g., low-flow oxygen, high-flow oxygen, non-invasive ventilation), and duration of oxygen supplementation. In accordance with the available structured dataset, severe disease was operationalized using objective treatment-based indicators of severity (need for supplemental oxygen and/or ICU admission).

Therapeutic variables extracted from the database included antiviral agents (remdesivir, favipiravir) and their treatment duration; immunomodulatory therapy (e.g., anakinra [Kineret^®^], when administered); systemic corticosteroid therapy and duration; antibiotic therapy and duration; and anticoagulation therapy (type, dose category when available, and duration).

Laboratory parameters recorded at admission and, when available, at discharge included leukocyte count, ESR, CRP, D-dimer, IL-6, procalcitonin, ferritin, ALT, AST, urea, creatinine, and fibrinogen. All analyses were performed in the central accredited laboratory of the “Victor Babeș” Clinical Hospital of Infectious Diseases and Pneumophthisiology, Timișoara, Romania.

Complete blood count, including leukocyte count, was measured using an automated hematology analyzer (Sysmex XN-1000, Sysmex Corporation, Kobe, Japan). CRP concentrations were determined by an immunoturbidimetric assay on a Cobas Integra 400 Plus analyzer (Roche Diagnostics, Mannheim, Germany). IL-6 levels were quantified using an electrochemiluminescence immunoassay (Elecsys^®^ IL-6, Roche Diagnostics) on a Cobas e601 analyzer (Roche Diagnostics, Mannheim, Germany). D-dimer concentrations were assessed by immunoturbidimetric assay (STA^®^-Liatest D-Di, Diagnostica Stago, Asnières-sur-Seine, France) on the STA Compact Max analyzer (Diagnostica Stago, France). Serum creatinine and liver enzymes (ALT, AST) were measured using standard kinetic or enzymatic methods on the Cobas Integra 400 Plus analyzer (Roche Diagnostics, Mannheim, Germany).

CT pulmonary angiography (CTPA) was performed at the discretion of treating physicians, guided by clinical suspicion of pulmonary embolism (e.g., unexplained hypoxemia, sudden clinical deterioration, elevated D-dimer). Only patients undergoing CTPA were eligible for PE adjudication, and PE incidence therefore reflects the subset of imaged patients rather than the entire ward population.

Admission values were defined as the earliest measurements obtained during the initial 24 h of hospitalization. Biomarkers were analyzed primarily as continuous variables and, when clinically appropriate, were also categorized using predefined cut-offs or distribution-based thresholds to account for potential non-linear associations with outcome

### 2.4. Exposure Definition: Diabetes Mellitus

Diabetes Mellitus (DM) was defined as present if the dataset recorded type 1 diabetes, type 2 diabetes, or newly diagnosed diabetes during the index hospitalization. Patients without entries in any of these specific diabetes-related variables were classified as non-DM.

If a specific diabetes type and “newly diagnosed diabetes” were both documented, classification relied on the explicit diabetes type (type 1 or type 2), while the “newly diagnosed” label denoted incident diabetes identified during that admission. Diabetes status was thus ascertained from structured electronic fields and physician documentation (including diagnosis coding and antidiabetic treatment records), rather than self-report alone.

### 2.5. Cardiovascular Variables and Definitions

Given the study’s focus on cardiovascular comorbidity burden and acute cardiovascular events, cardiovascular status was evaluated in two complementary dimensions.

(A) Baseline cardiovascular comorbidity burden: Pre-existing cardiovascular disease was defined by documentation of one or more of the following comorbidities in structured fields: arterial hypertension, ischemic heart disease, atrial fibrillation, prior ischemic stroke, left ventricular hypertrophy, or other physician-documented cardiovascular conditions.

(B) In-hospital cardiovascular and thromboembolic complications: A composite endpoint reflecting acute in-hospital cardiovascular and thromboembolic complications was constructed and included: Documented pulmonary embolism (PE), with location and whether PE led to ICU transfer, where available; Cardiorespiratory arrest; New ischemic changes documented during hospitalization, as captured in the dataset (including electrocardiographic and/or clinical evidence consistent with acute ischemia).

Thrombolytic therapy, when recorded, was included as a surrogate marker of life-threatening thromboembolic events but interpreted cautiously given its nature as a therapeutic intervention rather than a diagnosis-based endpoint. All definitions were prespecified in accordance with the structured data architecture to ensure consistent and reproducible case ascertainment.

### 2.6. Outcomes

The primary outcome was in-hospital all-cause mortality, defined as death from any cause occurring during the index hospitalization, as documented in the database.

Secondary outcomes included: ICU admission and ICU length of stay (when available). ICU admission followed institutional criteria, including need for invasive or non-invasive ventilatory support, hemodynamic instability, or other organ support requirements. These criteria were applied consistently across the study period, although individual triage decisions could also reflect contemporaneous resource availability. Requirement for any oxygen therapy and duration of oxygen supplementation. Total length of hospitalization (days). Occurrence of the in-hospital cardiovascular/thromboembolic composite endpoint described above.

### 2.7. Statistical Analysis

All statistical analyses were performed using IBM SPSS Statistics, version 26 (IBM Corp., Armonk, NY, USA). Continuous variables were assessed for distributional assumptions using histograms and Q–Q plots, complemented by formal normality testing where appropriate (e.g., Shapiro–Wilk test). Continuous variables are reported as mean ± standard deviation (SD) for approximately normally distributed data or median (interquartile range, IQR) for non-normally distributed data, and categorical variables as counts and percentages.

Between-group comparisons (DM vs. non-DM) were performed using independent-samples *t*-tests for normally distributed continuous variables and Mann–Whitney U tests for non-normally distributed variables. Categorical variables were compared using chi-square tests or Fisher’s exact tests when expected cell counts were small.

To evaluate whether DM was independently associated with adverse outcomes, multivariable logistic regression models were constructed. The primary multivariable model used in-hospital mortality as the dependent variable, whereas secondary models assessed ICU admission and/or the cardiovascular/thromboembolic composite endpoint, where event counts allowed reliable modelling. Given the limited number of events, we additionally fitted a parsimonious model including diabetes, age, sex, BMI, and admission creatinine only, as a sensitivity analysis to reduce the risk of overfitting. Covariates were selected a priori based on clinical relevance and dataset availability, including age, sex, BMI, smoking status, vaccination status, and baseline cardiovascular comorbidity burden. Calendar period (early vs. later pandemic phases) was explored descriptively, but was not included as a covariate in multivariable models because of limited events-per-variable and concern that further increasing model complexity would exacerbate overfitting. Laboratory markers (e.g., CRP, D-dimer, IL-6, creatinine) were included based on clinical plausibility and data completeness, with attention to limiting model complexity to reduce the risk of overfitting. Admission creatinine was included as a marker of baseline renal dysfunction, acknowledging that renal impairment may lie on the causal pathway between diabetes and adverse COVID-19 outcomes. To explore potential mediation and overadjustment, we pre-specified sensitivity models excluding admission creatinine from the adjustment set. Its inclusion therefore carries a risk of overadjustment and potential mediation bias, which we addressed in sensitivity analyses. Model assumptions and diagnostics included assessment of multicollinearity and evaluation of goodness of fit (e.g., Hosmer–Lemeshow test, where applicable).

Results are presented as odds ratios (ORs) with 95% confidence intervals (CIs). For the primary outcome (in-hospital mortality), 29 events occurred overall (9 in patients with DM and 20 in non-DM), corresponding to approximately 3–4 events per variable in the multivariable model including diabetes and nine covariates, i.e., clearly below commonly recommended thresholds for logistic regression stability. For ICU admission (21 events overall, 7 in DM and 14 in non-DM), the events-per-variable were around 3, also indicating a high risk of model overfitting and unstable estimates. All statistical tests were two-sided, and *p* < 0.05 was considered statistically significant.

Missing data were handled using an available-case approach (pairwise exclusion per analysis). Denominators for each variable are reflected through valid percentages in SPSS outputs and are explicitly reported in tables where appropriate.

### 2.8. Ethics

The study protocol was approved by the local Ethics Committee of the “Victor Babeș” Clinical Hospital for Infectious Diseases and Pneumophthisiology, Timișoara, Romania (approval no. 278/approval date: 15 January2026). The study was conducted using routinely collected clinical data, and ethics approval in January 2026 therefore represented a retrospective approval for analysis and publication of anonymized data from the predefined study period (March 2020 to December 2024). Written informed consent was obtained from all subjects involved in the study. All data were anonymized prior to analysis.

## 3. Results

### 3.1. Study Population

Between March 2020 and December 2024, 395 consecutive adult patients hospitalized with laboratory-confirmed SARS-CoV-2 infection were included in the final analysis. The temporal distribution of admissions mirrored the major pandemic phases, with a large proportion of cases during the 2020–2021 pre-vaccination and early-vaccination waves and additional admissions in 2022–2024, when broader population-level immunity and guideline-concordant standard-of-care regimens (including systemic corticosteroids for hypoxemic patients and IL-6 receptor antagonists in selected cases) had been widely implemented. Diabetes mellitus (DM) was present in 98/395 patients (24.8%), while 297/395 patients (75.2%) were classified as non-DM.

Within the DM cohort (*n* = 98), diabetes categories were distributed as follows: type 2 diabetes (T2DM), 63/98 (64.3%); newly diagnosed diabetes, 20/98 (20.4%); and type 1 diabetes (T1DM), 15/98 (15.3%), as shown in Table 1

### 3.2. Baseline Demographic and Clinical Characteristics (DM vs. Non-DM)

Baseline characteristics are summarized in Table 1. Mean age was similar between groups (71.69 ± 9.95 years in DM vs. 71.66 ± 11.78 years in non-DM; *p* = 0.979). Sex distribution did not differ (male sex: 53/98 [54.1%] vs. 160/297 [53.9%]; *p* = 1.000). Residence was comparable (urban: 55/98 [56.1%] vs. 153/297 [51.5%]; *p* = 0.499). Vaccination status was also similar between cohorts (vaccinated: 61/98 [62.2%] vs. 201/297 [67.7%]; *p* = 0.388). Smoking was numerically less frequent among DM patients but did not reach statistical significance (smokers: 27/98 [27.6%] vs. 107/297 [36.0%]; *p* = 0.157). Body mass index (BMI) showed a non-significant trend toward higher values in DM patients (median BMI 29.3 [25.3–32.8] vs. 27.9 [23.4–32.2]; *p* = 0.072).

Regarding cardiovascular comorbidity burden, arterial hypertension (binary derived from “HTA GRD”) was highly prevalent in both groups (HTA: 82/98 [83.7%] vs. 230/297 [77.4%]; *p* = 0.242). Pre-existing ischemic heart disease, atrial fibrillation, and prior ischemic stroke were not significantly different between DM and non-DM: Ischemic heart disease: 21/98 (21.4%) vs. 54/297 (18.2%); *p* = 0.574; Atrial fibrillation: 18/98 (18.4%) vs. 50/297 (16.8%); *p* = 0.846; Prior ischemic stroke: 28/98 (28.6%) vs. 66/297 (22.2%); *p* = 0.253.

### 3.3. In-Hospital Outcomes: Mortality, ICU Admission, and Length of Stay

Overall, in-hospital mortality was 29/395 (7.3%), as in Table 2. Mortality was numerically higher in DM patients compared with non-DM patients (9/98 [9.2%] vs. 20/297 [6.7%]), without statistical significance (*p* = 0.560). ICU admission occurred in 21/395 (5.3%) patients, again slightly more frequent in DM (7/98 [7.1%]) than non-DM (14/297 [4.7%]), without significance (*p* = 0.503). Median length of hospitalization was comparable between groups: 10 days (IQR 7–18) in DM vs. 10 days (IQR 7–16) in non-DM (*p* = 0.485).

Exploratory comparisons of crude outcomes across early (2020–2021) versus later (2022–2024) admission periods did not reveal obvious qualitative differences by diabetes status, but subgroup numbers were too small to support formal era-stratified modelling.

### 3.4. Pulmonary Embolism on CT Pulmonary Angiography

CT pulmonary angiography (CTPA) was performed in approximately 150/395 patients overall (~38%). Among those undergoing CTPA, pulmonary embolism was confirmed in 47 patients (≈31% of imaged patients; 11.9% of the total cohort). PE positivity did not differ by diabetes status (13/98 [13.3%] in DM vs. 34/297 [11.4%] in non-DM; *p* = 0.763; Table 2). Given that CTPA was reserved for patients with clinical or laboratory features suggestive of PE (e.g., unexplained hypoxemia, sudden deterioration, elevated D-dimer), these findings must be interpreted in the context of selection by indication and cannot be extrapolated to the entire hospitalized population. According to institutional protocols, indications and decision thresholds for CTPA (e.g., unexplained hypoxemia, sudden clinical deterioration, markedly elevated D-dimer) were applied consistently throughout the study period and did not differ systematically between patients with and without diabetes.

Within the cardiovascular/thromboembolic composite endpoint, individual component events occurred infrequently. Pulmonary embolism accounted for the majority of recorded events, whereas cardiorespiratory arrest and new ischemic changes were rare, precluding robust component-specific analyses and suggesting that no single component clearly dominated the composite in terms of frequency.

### 3.5. Admission Laboratory Findings (DM vs. Non-DM)

Admission biomarkers are detailed in Table 2, while a full panel of inflammatory and coagulation markers is provided in Supplementary Table S1 to improve readability. Oxygen saturation at presentation (SpO_2_) was modestly higher in DM patients (median 91.3 [90.0–94.6] vs. 90.6 [89.0–92.5]; *p* = 0.004). The absolute difference was small, and in light of subsequent analyses, is more likely related to differences in admission thresholds for high-risk patients than to true differences in respiratory involvement.

Inflammatory biomarkers were broadly comparable between groups: CRP: 106.9 [40.4–139.5] vs. 109.6 [55.3–145.0]; *p* = 0.227; IL-6: 14.31 [5.00–33.83] vs. 10.49 [4.39–31.66]; *p* = 0.331; Procalcitonin: 0.39 [0.15–0.90] vs. 0.40 [0.15–0.90]; *p* = 0.793; Leukocytes: 10.95 [7.75–15.32] vs. 11.91 [8.08–15.30]; *p* = 0.350. Coagulation markers at admission did not differ: D-dimer: 0.72 [0.54–1.17] vs. 0.76 [0.51–1.10]; *p* = 0.980; Fibrinogen: 3.65 [3.01–4.23] vs. 3.65 [3.01–4.24]; *p* = 0.972; PT-INR: 1.07 [0.98–1.16] vs. 1.06 [0.98–1.16]; *p* = 0.795; aPTT: 33.2 [29.6–36.4] vs. 33.2 [29.6–36.4]; *p* = 0.556. Renal function markers demonstrated a clinically relevant difference consistent with higher baseline renal vulnerability in DM patients: Urea: 55.6 [41.0–79.1] vs. 48.6 [39.2–68.0]; *p* = 0.047; Creatinine: 1.04 [0.76–1.52] vs. 0.88 [0.59–1.33]; *p* = 0.008. Admission lactate values were similar (lactate: 1.6 [1.3–2.0] vs. 1.6 [1.3–2.0]; *p* = 0.821).

### 3.6. Diabetes-Related Metabolic Parameters

Admission glucose and chronic glycemic control indicators (e.g., HbA1c) were not available for analysis in the dataset, precluding stratification by acute hyperglycemia, stress dysglycemia, or long-term metabolic control and limiting our ability to distinguish whether risk was primarily driven by chronic diabetes status or by acute glycemic derangements. This limitation substantially constrains mechanistic interpretation and restricts comparability with prior studies that have emphasized the prognostic role of hyperglycemia and chronic glycemic control in COVID-19. Earlier admission of high-risk patients with diabetes at slightly higher saturation thresholds could attenuate crude differences in outcomes and partially dilute observed associations between diabetes and adverse in-hospital events.

### 3.7. Regression Analyses: Association of Diabetes with Mortality and ICU Admission

Univariable logistic regression showed no significant association between DM and in-hospital mortality (OR 1.40, 95% CI 0.62–3.19, *p* = 0.422) or ICU admission (OR 1.55, 95% CI 0.61–3.97, *p* = 0.356) (Table 3).

**Figure 1:**
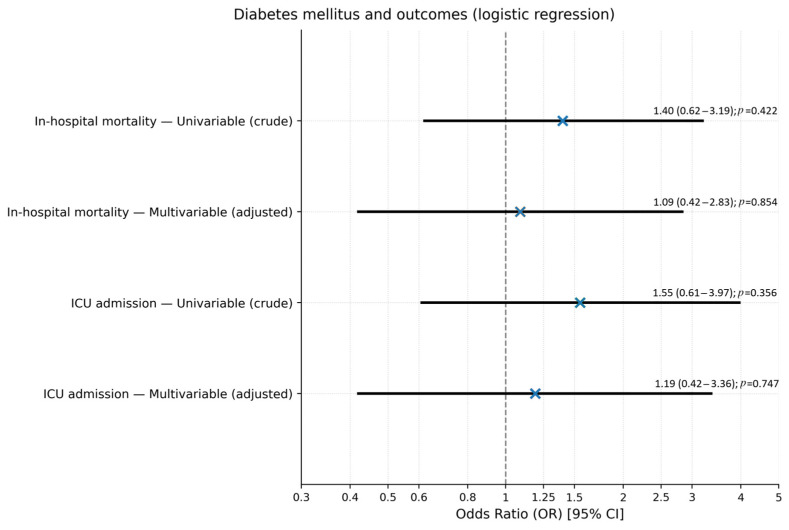
Association between diabetes mellitus and in-hospital mortality and ICU admission in multivariable logistic regression models. Models are adjusted for age, sex, BMI, vaccination status, arterial hypertension, ischemic heart disease, atrial fibrillation, prior ischemic stroke, and admission creatinine; non-DM patients constitute the reference category. Exact odds ratios with 95% confidence intervals and *p*-values are displayed next to each point estimate for ease of interpretation. The vertical dashed line indicates the null value (OR = 1), representing no association between diabetes mellitus and the outcomes.

In multivariable models adjusted for age, sex, BMI, vaccination status, hypertension, ischemic heart disease, atrial fibrillation, prior ischemic stroke, and admission creatinine, DM remained not independently associated with mortality (adjusted OR 1.09, 95% CI 0.42–2.83, *p* = 0.854) or ICU admission (adjusted OR 1.19, 95% CI 0.42–3.36, *p* = 0.747), as shown in Figure 1 and Table 3.

To explore potential overadjustment through renal function, we repeated the multivariable models excluding admission creatinine from the adjustment set. In these sensitivity analyses, diabetes remained not statistically associated with in-hospital mortality or ICU admission, but odds ratios were numerically higher and confidence intervals remained wide, underlining limited precision and possible residual mediation effects.

In a parsimonious sensitivity model adjusted only for age, sex, BMI, and admission creatinine, diabetes likewise showed no statistically significant association with in-hospital mortality or ICU admission, with odds ratios of similar magnitude and similarly wide confidence intervals. This supports the robustness of the direction of the estimates but does not resolve the limited precision due to small event counts.

## 4. Discussion

### 4.1. Principal Findings

In this single-center real-world cohort of 395 consecutively hospitalized adults with laboratory-confirmed SARS-CoV-2 infection, diabetes mellitus was present in nearly one-quarter of patients (24.8%). The principal findings were: (i) in-hospital mortality was numerically higher in diabetic patients (9.2% vs. 6.7%), but without statistical significance; (ii) ICU admission was also numerically more frequent in diabetic patients (7.1% vs. 4.7%), without statistical significance; (iii) pulmonary embolism positivity on CTPA did not differ by diabetes status; (iv) admission inflammatory and coagulation biomarkers were broadly comparable between groups; and (v) renal function markers (urea and creatinine) were significantly higher among diabetic patients, indicating increased baseline renal vulnerability. In adjusted regression models, diabetes was not statistically associated with in-hospital mortality or ICU admission; nonetheless, these null findings must be interpreted in the context of limited statistical power and lack of detailed glycemic information.

### 4.2. Diabetes and COVID-19 Outcomes: Association vs. Independence After Adjustment

Since the earliest pandemic waves, diabetes has been consistently reported as a major risk factor for severe COVID-19 and death in large population-based datasets. In the OpenSAFELY platform analysis of linked electronic health records in England, diabetes emerged as one of the conditions associated with increased COVID-19-related mortality, alongside older age, male sex, socioeconomic deprivation, and cardiovascular/renal comorbidities [17]. Consistently, nationwide studies focusing specifically on diabetes populations showed higher COVID-19 mortality in both type 1 and type 2 diabetes during the first wave in England [18].

However, the extent to which diabetes represents an independent predictor (as opposed to a marker of clustered vulnerability) varies substantially across study designs. In hospital-based cohorts, diabetes-associated excess risk may be attenuated after adjustment for age, baseline renal dysfunction, cardiovascular disease burden, and markers of acute severity. Our findings are consistent with a risk-marker paradigm: despite modest numerical differences in crude outcomes, diabetes was not statistically associated with in-hospital death or ICU admission after multivariable adjustment. However, given the small number of events and wide confidence intervals, our data cannot exclude a clinically relevant independent effect of diabetes. This suggests that in real-world hospitalized populations, diabetes may contribute to risk predominantly through accompanying organ dysfunction and comorbidity clustering rather than acting as a stand-alone driver of acute COVID-19 mortality. Therefore, the absence of statistically significant associations in our models should be interpreted as a consequence of limited power and model instability rather than as definitive evidence of no independent effect of diabetes.

### 4.3. Thromboinflammation and Pulmonary Embolism: No Diabetes-Specific Excess Risk

Severe COVID-19 is characterized by a prototypical thromboinflammatory phenotype, involving endothelial injury, platelet activation, immunothrombosis, and microvascular occlusion. Early clinical evidence demonstrated that abnormal coagulation parameters are associated with worse prognosis in COVID-19, supporting the central role of COVID-19-associated coagulopathy in severe disease trajectories [19]. Despite the well-established prothrombotic background in diabetes, our cohort did not show significant differences in PE positivity on CTPA between diabetic and non-DM patients. This finding is consistent with evidence suggesting that PE occurrence in COVID-19 is primarily driven by acute disease severity and thromboinflammatory activation rather than diabetes status alone, with imaging-based PE cohorts reporting heterogeneous risk patterns across patient subgroups [20]. Additionally, admission coagulation biomarkers (D-dimer, fibrinogen, INR, aPTT) were comparable across groups. This suggests that among hospitalized patients treated according to institutional anticoagulation protocols, thromboembolic risk may be largely driven by acute COVID-19 disease biology and severity rather than diabetes status itself.

COVID-19 is further complicated by a delicate risk–benefit balance between thrombotic events (e.g., DVT/PE) and major bleeding manifestations, including spontaneous hematomas, particularly in patients receiving anticoagulant therapy [21].

The low absolute frequency of individual cardiovascular and thromboembolic events, and the heterogeneity of components within the composite endpoint, further limit our ability to define diabetes-specific effects on particular event types.

### 4.4. Inflammatory Response: Similar Biomarker Profiles at Admission

Hyperinflammation and cytokine signaling play a central role in severe COVID-19, with IL-6 representing a major mediator of immunopathology and clinical progression. The clinical relevance of IL-6 biology has been supported by randomized evidence demonstrating improved survival and other outcomes with IL-6 receptor blockade in selected hospitalized COVID-19 patients with systemic inflammation [22]. Observational data further indicate that IL-6—particularly when interpreted together with cardiac biomarkers—can discriminate risk in sepsis-like COVID-19 trajectories, reinforcing its role as a clinically actionable marker of systemic inflammation rather than a diabetes-specific signal [23,24]. Although diabetes is associated with chronic low-grade inflammation, we observed broadly comparable admission inflammatory profiles (CRP, IL-6, procalcitonin, leukocyte count) between diabetic and non-DM patients. This absence of a markedly amplified inflammatory signature may partly explain why diabetes did not remain independently associated with clinical outcomes in adjusted models. Finally, although our study focused primarily on cardiometabolic and thromboinflammatory determinants, secondary bacterial infections—including bloodstream infections—represent an additional severity amplifier in hospitalized COVID-19 that may interact with diabetes-related immune dysregulation, contributing to prolonged hospitalization and worse outcomes in selected patients [25].

Obesity and metabolic syndrome represent additional cardiometabolic vulnerability phenotypes in COVID-19, with evidence linking these conditions to diastolic dysfunction trajectories in the post-COVID setting, even among apparently healthy individuals. These observations support the concept that cardiometabolic burden extends beyond diabetes alone and may influence resilience during acute infection [26].

Emerging evidence also implicates gut microbiome dysbiosis as a modulator of systemic inflammation in COVID-19, potentially influencing immune responses and cardiometabolic vulnerability pathways, although this relationship remains insufficiently characterized in routine hospital cohorts [27].

### 4.5. Renal Vulnerability as the Most Consistent Diabetes-Associated Signal

A consistent differentiating feature was renal function impairment: diabetic patients had significantly higher urea and creatinine at admission. This observation is clinically important, as kidney dysfunction is a recognized determinant of COVID-19 severity and mortality and may act as both mediator and effect modifier in diabetes-associated risk pathways [28,29]. From a causal perspective, renal dysfunction is likely to act at least partly as a mediator of the excess risk associated with diabetes rather than as a pure confounder. Adjusting for creatinine may therefore attenuate the apparent effect of diabetes and bias estimates toward the null. Our sensitivity analyses excluding creatinine from the adjustment set yielded similar qualitative findings but cannot fully resolve this mediation issue. Diabetes is also one of the leading contributors to chronic kidney disease and subclinical renal impairment, which may reduce physiologic reserve during acute systemic infection. The prominence of renal vulnerability in our cohort supports the concept that diabetics constitute a higher-risk phenotype primarily through renal–cardiometabolic pathways rather than solely through metabolic dysregulation.

### 4.6. Oxygenation at Presentation and Admission Thresholds in High-Risk Patients

Unexpectedly, diabetic patients presented with slightly higher admission oxygen saturation compared with non-DM patients. The absolute difference was modest, and the most plausible explanation is not a protective physiological effect but rather healthcare system dynamics, including earlier hospitalization thresholds, differential referral patterns, and greater physician vigilance for high-risk patients with diabetes. This likely reflects admission bias rather than a true difference in underlying respiratory involvement.

### 4.7. Clinical Implications

In hospitalized COVID-19 patients, diabetes status should be interpreted within an integrated vulnerability framework. Our results suggest that risk stratification should prioritize objective severity and organ dysfunction markers—particularly renal impairment—rather than using diabetes status alone as a determinant of intensive monitoring or escalation. This approach aligns with contemporary guideline-driven COVID-19 care, which emphasizes severity phenotyping and dynamic clinical progression.

### 4.8. Strengths and Limitations

Strengths of this study include consecutive inclusion of all adult patients hospitalized with laboratory-confirmed SARS-CoV-2 infection over nearly five years (March 2020–December 2024) at a single tertiary infectious diseases institution. This design reflects actual inpatient pathways in an Eastern European setting. It captures temporal heterogeneity in circulating variants, vaccination uptake, and evolving management strategies.

A major strength is the prospectively maintained, standardized departmental database. It systematically records patient demographics, comorbidities, cardiovascular history, biomarkers, imaging, therapeutic exposures, and hard in-hospital outcomes. This enabled consistent data extraction, outcome assessment, and reliable multivariable adjustment, including vaccination status and organ dysfunction markers.

This cohort provides detailed hospital-level phenotyping that many administrative datasets lack. It jointly characterizes diabetes status, cardiovascular burden, thromboinflammatory markers, and renal vulnerability. The observed higher admission urea/creatinine in diabetics, and loss of independent diabetes associations with severe outcomes after adjustment, support a risk-marker paradigm in an underrepresented regional context. Compared with earlier reports using limited panels, detailed biomarker profiling and systematically adjudicated CTPA-confirmed PE offered a strong integrated assessment. Clinically relevant outcomes were evaluated using prespecified multivariable models adjusting for key confounders.

Several limitations should be acknowledged. First, the retrospective, single-center design limits external generalizability. Admission thresholds, therapeutic algorithms, and resource availability in a tertiary infectious diseases center may differ from those in other settings. Residual confounding by unmeasured factors (e.g., socioeconomic status, diabetes duration, outpatient regimens, and adherence) cannot be excluded despite comprehensive capture of major comorbidities.

Second, the overall sample size (*n* = 395) and modest event counts (deaths, *n* = 29; ICU admissions, *n* = 21) limit statistical power to detect small-to-moderate independent effects and compromise model stability. As detailed in the Methods, the events-per-variable in our multivariable models were approximately 3–4 for mortality and around 3 for ICU admission, clearly below commonly recommended thresholds for logistic regression stability. Consequently, the low events-per-variable in our multivariable logistic regression models increases the risk of overfitting and false-negative findings, particularly for moderate independent effects of diabetes.

A further major limitation is that key glycemic indices were unavailable. Admission glucose, HbA1c, in-hospital glycemic trajectories, diabetes duration, diabetes-specific complications, and outpatient glucose-lowering therapies were not systematically collected. As a result, our study essentially evaluates a binary diabetes label rather than glycemic status per se and cannot address whether acute or chronic dysglycemia independently drives risk. Because of very small numbers of type 1 and newly diagnosed diabetes, sensitivity analyses excluding these subgroups were not feasible, and this should be considered when interpreting our findings. This severely constrains mechanistic interpretation and limits comparability with prior studies that have demonstrated strong associations between hyperglycemia, poor chronic control, and adverse COVID-19 outcomes, even within diabetic populations.

Another important consideration is that biomarker analyses were largely limited to admission values, without longitudinal profiling of inflammation, coagulation, and renal function. Missing data were handled pragmatically via available-case analysis. This may introduce bias if missingness is severity-related and reduces the effective sample size for some analyses.

We did not formally adjust for calendar period in regression models, as including additional temporal covariates would have further reduced the events-per-variable and increased the risk of overfitting.

Lastly, although the cohort spans multiple pandemic years, the analysis did not stratify granularly by viral variants, pandemic waves, or key standard-of-care changes, including corticosteroid exposure and the use of IL-6 receptor antagonists. These time-varying factors may act as important effect modifiers. Consequently, our findings may be partly era-specific and should be interpreted with caution when extrapolating to other periods and healthcare systems, given the evolving thromboinflammatory biology and treatment landscape of COVID-19 [30,31,32,33].

## 5. Conclusions

In this single-center Eastern European cohort of consecutively hospitalized adults with COVID-19, diabetes mellitus was frequent and associated with a higher burden of cardiovascular comorbidities and impaired renal function at admission. After adjustment for measured comorbidities, vaccination status, and renal markers, diabetes was not statistically associated with in-hospital mortality or ICU admission. Nevertheless, the limited number of outcome events, the low events-per-variable in multivariable models, and the absence of detailed glycemic indices restrict our ability to draw firm conclusions regarding the independent prognostic role of diabetes. In addition, the absence of admission glucose, HbA1c, and in-hospital glycemic trajectories further limits our ability to assess the independent prognostic role of dysglycemia. Clinically, these findings support viewing diabetes as a marker of clustered cardiometabolic and renal vulnerability, reinforcing the need to focus on objective severity and organ dysfunction markers while still recognizing diabetic patients as a high-risk group requiring close monitoring and optimized care.

## Figures and Tables

**Table 1:** Baseline demographic and clinical characteristics of hospitalized patients with SARS-CoV-2 infection according to diabetes mellitus status.

Variable	Diabetes Mellitus (*n* = 98)	Non-Diabetes Mellitus (*n* = 297)	*p*-Value
Age (years)—mean ± SD	71.69 ± 9.95	71.66 ± 11.78	0.979
Sex—male *n* (%)	53 (54.1%)	160 (53.9%)	1.000
Place of residence—urban *n* (%)	55 (56.1%)	153 (51.5%)	0.499
Vaccinated against SARS-CoV-2 *n* (%)	61 (62.2%)	201 (67.7%)	0.388
Smoking status—current/former smoker *n* (%)	27 (27.6%)	107 (36.0%)	0.157
BMI (kg/m^2^)—median [IQR]	29.3 [25.3–32.8]	27.9 [23.4–32.2]	0.072
Diabetes type (only in DM group)			
Type 2 diabetes	63 (64.3%)	—	—
Newly diagnosed diabetes	20 (20.4%)	—	—
Type 1 diabetes	15 (15.3%)	—	—
Cardiovascular comorbidities			
Arterial hypertension	82 (83.7%)	230 (77.4%)	0.242
Ischemic heart disease	21 (21.4%)	54 (18.2%)	0.574
Atrial fibrillation	18 (18.4%)	50 (16.8%)	0.846
Prior ischemic stroke	28 (28.6%)	66 (22.2%)	0.253

Note: “—” Not applicable, as diabetes subtype classification was available only for patients with diabetes mellitus.

**Table 2:** In-hospital outcomes, pulmonary embolism and selected admission laboratory parameters according to diabetes mellitus status.

Variable	Diabetes Mellitus (*n* = 98)	Non-Diabetes Mellitus (*n* = 297)	*p*-Value
In-hospital mortality *n* (%)	9 (9.2%)	20 (6.7%)	0.560
ICU admission *n* (%)	7 (7.1%)	14 (4.7%)	0.503
Length of hospitalization (days)—median [IQR]	10 (IQR 7–18)	10 (IQR 7–16)	0.485
Pulmonary embolism on CTPA n (%)	13 (13.3%)	34 (11.4%)	0.763
Admission SpO_2_ (%)—median [IQR]	91.3 [90.0–94.6]	90.6 [89.0–92.5]	0.004
Admission renal function markers—median [IQR]			
Urea (mg/dL)	55.6 [41.0–79.1]	48.6 [39.2–68.0]	0.047
Creatinine (mg/dL)	1.04 [0.76–1.52]	0.88 [0.59–1.33]	0.008
Admission lactate (mmol/L)—median [IQR]	1.6 [1.3–2.0]	1.6 [1.3–2.0]	0.821

**Table 3:** Association between diabetes mellitus and key in-hospital outcomes: univariable and multivariable logistic regression results.

Outcome	Model	Odds Ratio (OR)	95% Confidence Interval (CI)	*p*-Value
In-hospital mortality	Univariable (crude)	1.40	0.62–3.19	0.422
	Multivariable (adjusted)	1.09	0.42–2.83	0.854
ICU admission	Univariable (crude)	1.55	0.61–3.97	0.356
	Multivariable (adjusted)	1.19	0.42–3.36	0.747

Notes: Univariable models include only diabetes mellitus (DM vs. non-DM) as predictor. Multivariable models are adjusted for the following covariates selected a priori based on clinical relevance and dataset availability: age, sex, BMI, vaccination status, arterial hypertension, ischemic heart disease, atrial fibrillation, prior ischemic stroke, and admission creatinine. All models were performed using logistic regression. Results are expressed as odds ratios (OR) with corresponding 95% confidence intervals (CI). A *p*-value < 0.05 was considered statistically significant. No significant independent association was found between diabetes mellitus and either in-hospital mortality or ICU admission after multivariable adjustment.

## Data Availability

Deidentified clinical, laboratory, and imaging data supporting the findings of this study are available from the corresponding authors upon reasonable request, subject to institutional data-sharing policies.

## Supplementary Materials

The following supporting information can be downloaded at: https://www.mdpi.com/article/10.3390/medicina62020410/s1, Table S1: Admission inflammatory and coagulation markers according to diabetes mellitus status.

## Abbreviations

The following abbreviations are used in this manuscript:

aPTT	activated partial thromboplastin time
ALT	alanine aminotransferase
AST	aspartate aminotransferase
BMI	body mass index
CBC	complete blood count
CKD	chronic kidney disease
COVID-19	coronavirus disease 2019
CRP	C-reactive protein
CTPA	computed tomography pulmonary angiography
DM	diabetes mellitus
D-dimer	fibrin degradation product (D-dimer)
ESR	erythrocyte sedimentation rate
FEU	fibrinogen equivalent units
HFNC	high-flow nasal cannula
HTA	arterial hypertension
ICU	intensive care unit
IHD	ischemic heart disease
IL-6	interleukin-6
IQR	interquartile range
LOS	length of stay
NIV	non-invasive ventilation
OR	odds ratio
PE	pulmonary embolism
PT-INR	prothrombin time—international normalized ratio
RT-PCR	reverse transcription polymerase chain reaction
SARS-CoV-2	severe acute respiratory syndrome coronavirus 2
SD	standard deviation
SpO_2_	peripheral oxygen saturation
T1DM	type 1 diabetes mellitus
T2DM	type 2 diabetes mellitus
