# Supplementary material for: Diabetes Mellitus and In-Hospital Outcomes in Hospitalized COVID-19 Patients: A Single-Center Eastern European Cohort Study (2020–2024)
Source: Medicina (Kaunas). 2026 Feb 21;62(2):410. doi: 10.3390/medicina62020410 (PMC12943625; doi:10.3390/medicina62020410)
Supplement: Supplementary file 1 [file medicina-62-00410-s001.zip › medicina-4136271-supplementary.pdf]

Supplementary Table S1. Admission inflammatory and coagulation markers according to diabetes mellitus status.

Admission inflammatory markers – median [IQR]			
CRP (mg/L)	106.9 [40.4 – 139.5]	109.6 [55.3 – 145.0]	0.227
IL-6 (pg/mL)	14.31 [5.00 – 33.83]	10.49 [4.39 – 31.66]	0.331
Procalcitonin (ng/mL)	0.39 [0.15 – 0.90]	0.40 [0.15 – 0.90]	0.793
Leukocytes (×10³/µL)	10.95 [7.75 – 15.32]	11.91 [8.08 – 15.30]	0.350
Admission coagulation markers – median [IQR]			
D-dimer (µg/mL FEU)	0.72 [0.54 – 1.17]	0.76 [0.51 – 1.10]	0.980
Fibrinogen (g/L)	3.65 [3.01 – 4.23]	3.65 [3.01 – 4.24]	0.972
PT-INR	1.07 [0.98 – 1.16]	1.06 [0.98 – 1.16]	0.795
aPTT (seconds)	33.2 [29.6 – 36.4]	33.2 [29.6 – 36.4]	0.556
